# Slip Effects on Mixed Convective Peristaltic Transport of Copper-Water Nanofluid in an Inclined Channel

**DOI:** 10.1371/journal.pone.0105440

**Published:** 2014-08-29

**Authors:** Fahad Munir Abbasi, Tasawar Hayat, Bashir Ahmad, Guo-Qian Chen

**Affiliations:** 1 Department of Mathematics, Quaid-I-Azam University, Islamabad, Pakistan; 2 Nonlinear Analysis and Applied Mathematics (NAAM) Research Group, Faculty of Science, King Abdulaziz University, Jeddah, Saudi Arabia; 3 College of Engineering, Peking University, Beijing, China; Texas A&M University, United States of America

## Abstract

Peristaltic transport of copper-water nanofluid in an inclined channel is reported in the presence of mixed convection. Both velocity and thermal slip conditions are considered. Mathematical modelling has been carried out using the long wavelength and low Reynolds number approximations. Resulting coupled system of equations is solved numerically. Quantities of interest are analyzed through graphs. Numerical values of heat transfer rate at the wall for different parameters are obtained and examined. Results showed that addition of copper nanoparticles reduces the pressure gradient, axial velocity at the center of channel, trapping and temperature. Velocity slip parameter has a decreasing effect on the velocity near the center of channel. Temperature of nanofluid increases with increase in the Grashoff number and channel inclination angle. It is further concluded that the heat transfer rate at the wall increases considerably in the presence of copper nanoparticles.

## Introduction

Mixed convective flows have received numerous attention from the researchers the world over due to their wide industrial and engineering applications. Convection is seen in the ocean currents, sea-wind formation, rising of plume of hot air from fire, formation of micro-structures during the cooling of molten metals, solar ponds and in fluid flows around heat dissipation fins. Convection in channels plays a vital role in heat exchangers, removal of nuclear waste and in modern cooling/heating systems. “Nanofluids” refer to heat transfer liquids with enhanced heat transfer capability. Such materials are obtained by suspending nanoparticles in traditional heat transfer liquids e.g. water, ethylene glycol and engine oil. A number of experimental studies elaborate that the convective heat transfer in nanofluids is considerably higher than that of the base fluids. Enhanced heat transfer in nanofluids has enabled there use in several electrical and engineering applications. This gives rise to a new branch of mechanics named nanofluid mechanics. The wide utility of nanofluids is the reason for growing interest in nanofluid mechanics by the researchers of modern era. The term nanofluid was introduced by Choi [1] in his effort to enhance the thermal conductivity of the fluids. Buongiorno [2] proposed that thermophoresis and Brownian motion are significant in the dynamics of nanofluids. Some studies using the Buongiorno's model of nanofluids can be seen through refs. [3]–[5]. Expressions for the effective density and viscosity of the nanofluids were computed by Xuan and Li [6]. Brinkman's [7] model for the effective viscosity of the two phase flow was found to be in good agreement with the experimental results (Xuan and Li. [8]). Tiwari and Das [9] proposed a model for the analysis of nanofluids employing the results of Xuan and Li [6] and Brinkman [7]. Model of Tiwari and Das [9] was used by many researchers for the analysis of nanofluid dynamics and was found in good agreement with the experimental data (see [10]–[13]).

Peristalsis is a well-known mechanism for fluid transport in physiology. In this mechanism sinusoidal waves travel on the walls of tube like organs of human beings propelling the fluid contained within the tube in the direction of their propagation. In physiology the principle of peristalsis is seen in the transport of food through oesophagus, movement of chyme in intestines, urine transport from kidneys to the bladder, bile transport in bile duct, transport of spermatozoa, vasomotion of blood vessels and many others. Peristalsis has proven very useful in fluid transport over short distance preventing the fluid from being contaminated. Subject to its utility, such mechanism has been adopted by the engineers in designing several industrial appliances including roller and finger pumps and peristaltic pumps in heart lung and dialysis machines. Latest hose pumps of several kinds operate through the principle of peristalsis. Peristaltic type flow is readily being used in the nuclear industry for the transport of corrosive fluids. Subject to such wide occurrence of peristalsis in physiology and industry many theoretical investigations are carried out in different flow configuration [14]–[22]. It is now well admitted fact that all the tubular organs facilitating fluid flow in human body are internally lubricated with mucus and secretion layers. These layers in turn prevent the fluid from sticking to the walls. In such cases the no-slip condition between the fluid and solid boundary is not valid. With such motivation some authors investigated the peristaltic transport of traditional fluids with slip effects [23]–[25].

It is noticed from existing information on the topic that not much has been said yet about the peristaltic motion of nanofluids. Few recent investigations constructed such flow models (see [26]–[27]). In these attempts, the Buongiorno's model of nanomaterial has been employed. However there is not a single attempt which can address the peristaltic transport of nanofluid employing Tiwari and Das [9] model. Intention in present communication is to prepare such fluid model describing peristalsis. Hence we consider here the copper-water nanofluid to discuss the peristaltic flow in a symmetric channel with slip conditions. The channel walls satisfy the velocity and thermal slip conditions. Inclined channel filled with an incompressible fluid is considered. Relevant modeling is presented. Computations for the governing problems are made numerically. Plots for different flow quantities of interest are displayed and analyzed.

## Formulation and Solution

**Figure 1:**
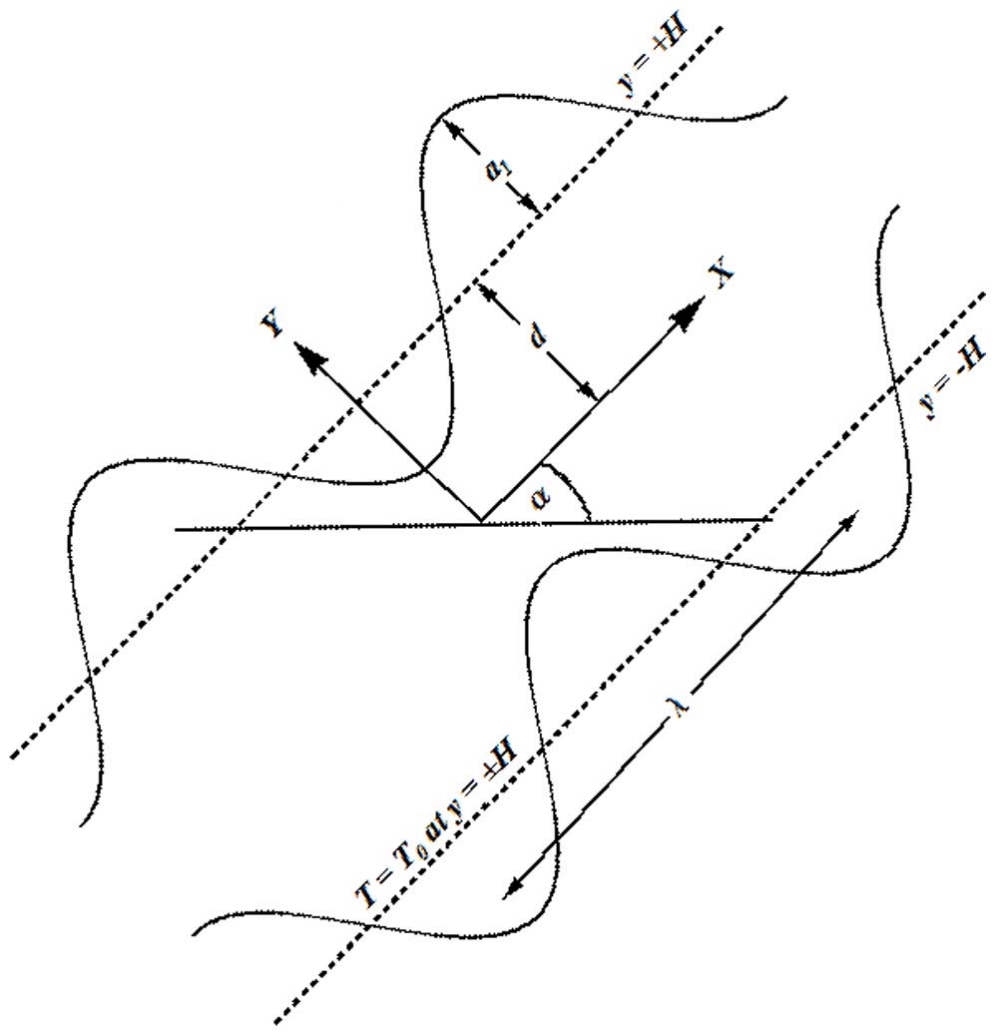
Geometry of the problem.

Consider the peristaltic flow of copper-water nanofluid in a symmetric channel of width 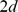. The channel is inclined at an angle 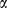 to the horizontal. Moreover the channel walls are maintained at constant temperature 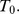 Cartesian coordinates are chosen in such a way that the 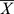-axis lies along the length of the channel whereas 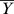 -axis is normal to 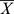 -axis. Waves of wavelength *λ* and amplitude 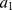 travel on the channel walls with constant speed 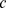. These waves are responsible for flow generation in the channel. Schematic diagram of the problem is given in Fig. 1.

Wave geometry is of the form
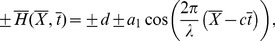
(1)where 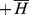 and 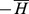 denote the upper and lower walls respectively. Velocity profile for such a flow is 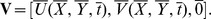 The two-dimensional continuity equation for an incompressible flow can be written as follows: 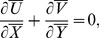
(2)where 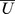 and 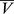 are the longitudinal and transverse components of velocity in the laboratory frame. The 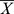 and 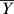 components of momentum equation in presence of mixed convection are
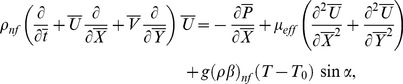
(3)

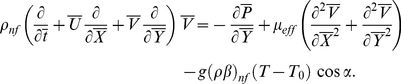
(4) Energy equation with viscous dissipation and heat generation/absorption is given by
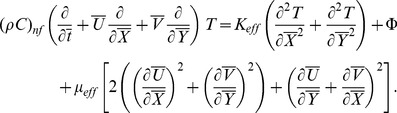
(5) In these equations 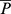 is the pressure in laboratory frame, 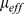
the effective viscosity of nanofluid, 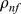 the density of nanofluid, 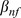 thermal expansion coefficient of the nanofluid, 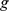 the acceleration due to gravity, 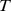 the fluid temperature, 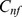 the specific heat of nanofluid, 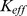 the effective thermal conductivity of nanofluid and 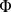 the dimensional heat source/sink parameter. Nomenclature of all the quantities is also provided through Table 1. Effective viscosity of the nanofluid can be computed with the help of equations for two-phase flow. In this study we have used the relation proposed by Brinkman [7]

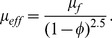
(6)


**Table 1:** Nomenclature.

Nomenclature			
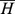 ( 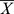 ,t)	Peristaltic wall	*d*	Half channel width
a_1_	Amplitude of peristaltic wave	*λ*	Wavelength of peristaltic wave
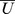	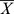 -component of velocity	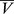	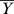 -component of velocity
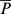	Pressure	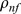	Density of nanofluid
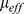	Effective viscosity	*g*	Acceleration due to gravity
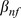	Thermal expansion coefficient of nanofluid	*α*	Channel inclination angle
T	Temperature	*T_0_*	Temperature of the walls
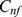	Specific heat of nanofluid	*K_eff_*	Effective thermal conductivity
Φ	Dimensional heat source/sink parameter	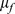	Viscosity of base fluid
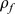	Density of base fluid	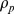	Density of nanoparticles
*C_f_*	Specific heat of base fluid	*C_P_*	Specific heat of nanoparticles
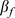	Thermal expansion coefficient of base fluid	*φ*	Nanoparticles volume fraction
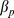	Thermal expansion coefficient of nanoparticle	*K_f_*	Thermal conductivity of base fluid
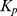	Thermal conductivity of nanoparticles	*E*	Eckert number
*Re*	Reynolds number	*Pr*	Prandtl number
*Gr*	Grashoff number	*Br*	Brinkman number
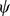	Stream function	*β*	Dimensionless velocity slip parameter
*F*	Dimensionless volume flow rate in wave frame	*γ*	Dimensionless thermal slip parameter
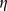	Dimensionless volume flow rate in fixed frame	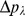	Pressure rise per wavelength

Relations for effective density, specific heat and thermal conductivity of nanofluid by Tiwari and Das [9] are
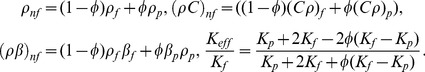
(7)Here Maxwell-Gamett's (MG-model) is used to approximate the effective thermal conductivity of the nanofluid. In these equations 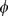 denotes the nanoparticle volume fraction, 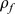 the density of base fluid (water in this case), 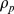 the density of nanoparticles, 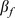 the thermal expansion coefficient of base fluid, 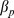 the thermal expansion of nanoparticles, 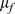 the viscosity of base fluid, 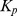 the thermal conductivity of nanoparticles and 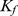 the thermal conductivity of base fluid. Numerical values of these properties are readily available which we include in the Table 2 [9], [10].

**Table 2:** Numerical values of the thermophysical properties.

Property	Basefluid/water	Nanoparticles/copper (Cu)
Density (kg/m^3^)	997.1	8933
Thermal conductivity (W/mK)	0.613	400
Specific heat (J/kgK)	4179	385
Thermal expansion coefficient (1/K)×10^−6^	210	16.65

The quantities from the laboratory frame 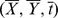 to wave frame 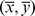 are related by the following transformations:
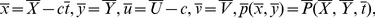
(8)in which 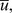 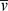 and 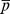 are the velocity components and pressure in wave frame (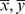). Equations (2)–(5) through Eqs. (6–8) yield
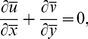
(9)

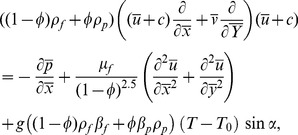
(10)

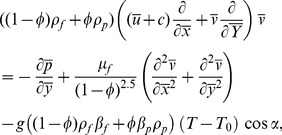
(11)

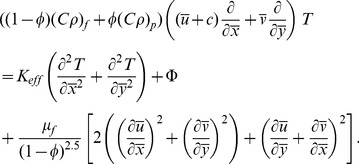
(12)With the help of following dimensionless quantities
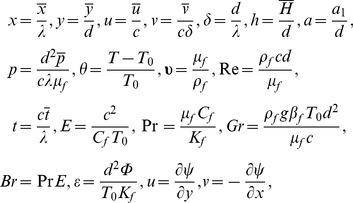
(13)and applying the long wavelength and low Reynolds number approximations Eqs. (10–12) are reduced to the following expressions:
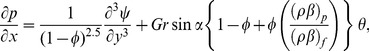
(14)

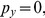
(15)

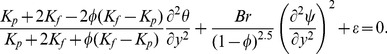
(16)where the continuity equation is identically satisfied and Eq. (15) shows that 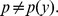 In these equations 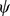
is the stream function, 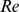 the Reynolds number, 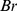 the Brinkman number, 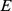 the Eckret number, 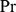 the Prandtl number, 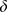 the wave number, 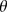 the dimensionless temperature and 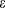 the dimensionless heat source/sink parameter. From Eqs. 14 and 15 one obtains
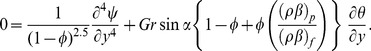
(17)Defining 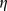 and F as the dimensionless mean flows in laboratory and wave frames by
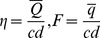
(18)two flow rates can be related as follows:
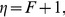
(19)where
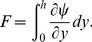
(20)The velocity and thermal slip conditions are
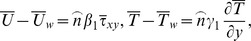
where 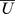 is the velocity of fluid, 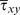 the component of extra stress tensor, 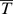 the temperature of fluid, 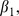 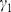 are respectively the dimensional velocity and thermal slip parameters and 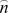 the unit normal on the wall. The dimensionless velocity and thermal slip conditions finally become
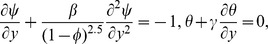
where 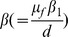 and 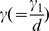 are respectively the dimensionless velocity and thermal slip pramaeters. The appropriate boundary conditions for the present flow model are
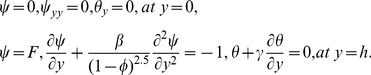
(21)with 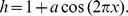 Pressure rise per wavelength (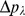) is defined as follows:
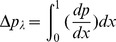
 The system of Eqs. (14–16) subject to boundary conditions (21) is solved numerically using NDSolve in Mathematica. We have taken the step size equal to 0.01 for the variations in both variables 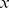 and 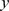. Obtained numerical results are analyzed graphically in the next section.

## Results and Discussion

Pressure gradient, pressure rise per wavelength, axial velocity and trapping are important quantities in the study of peristalsis. On the other hand the analysis of heat transfer phenomena is significant in the study of nanofluids. Hence the present section is prepared to analyze the behavior of pressure gradient, pressure rise per wavelength, axial velocity, streamlines, temperature and heat transfer rate at the wall for variation in different embedded parameters.

### Pressure gradient and pressure rise pre wavelength

**Figure 2:**
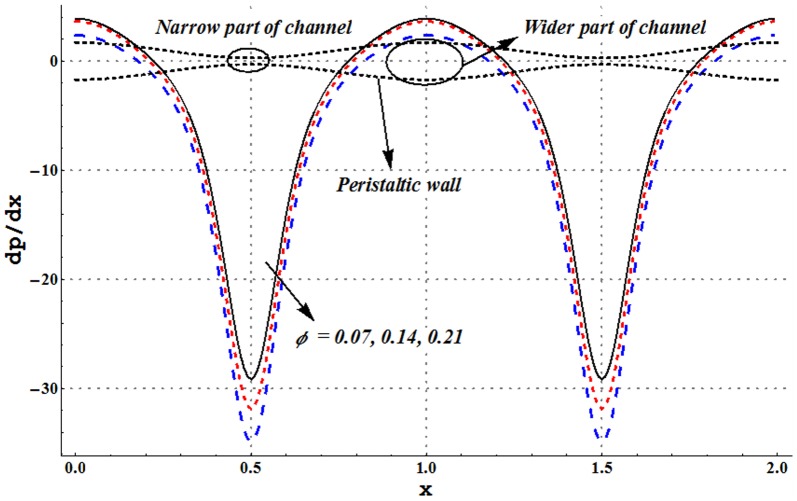
Pressure gradient for variation in nanoparticle volume fraction when 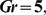 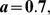 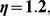 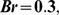 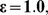 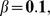 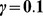 and 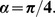

**Figure 3:**
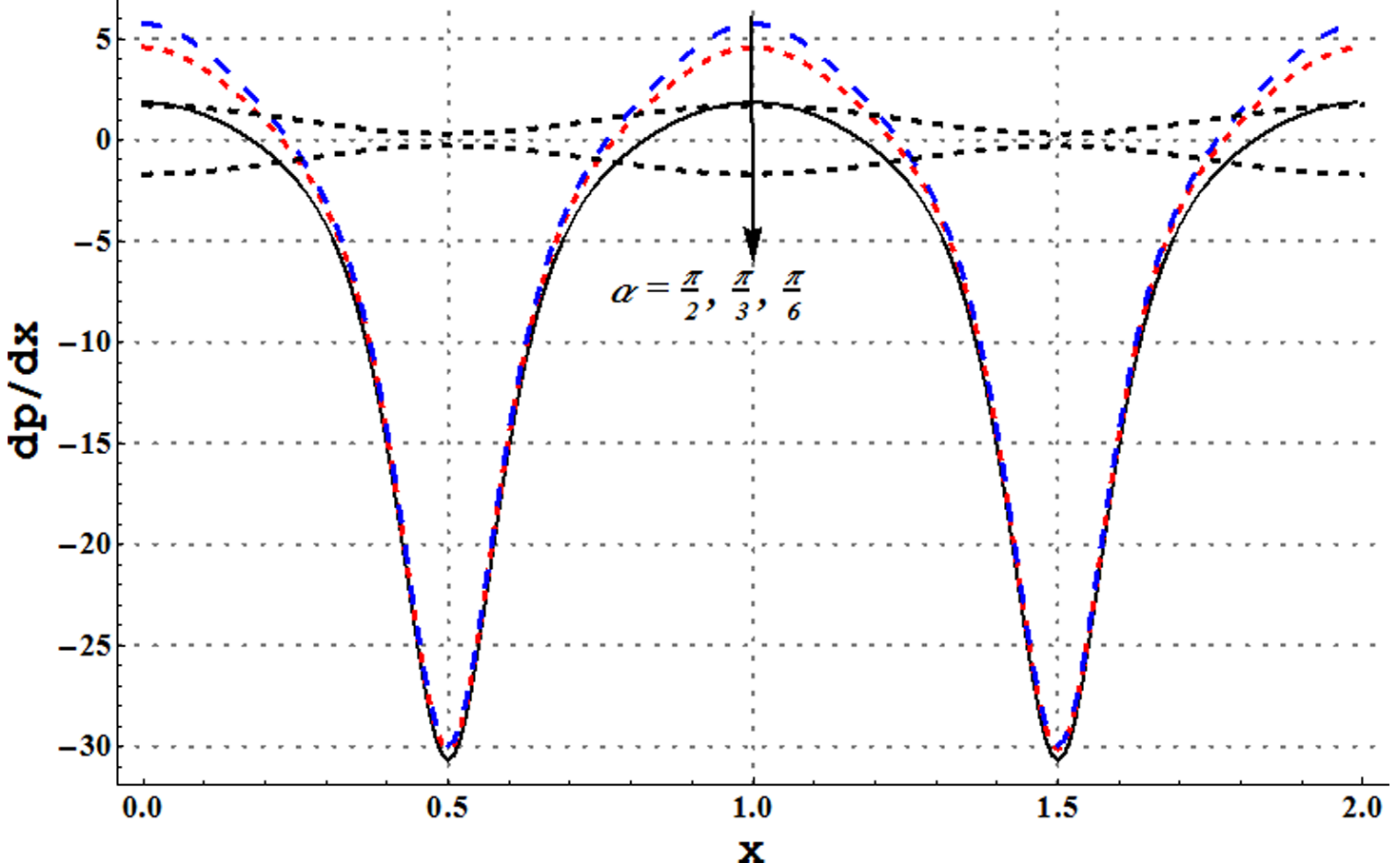
Pressure gradient for variation in channel inclination angle when 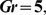 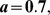 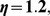 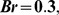 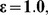 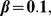 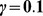 and 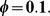

**Figure 4:**
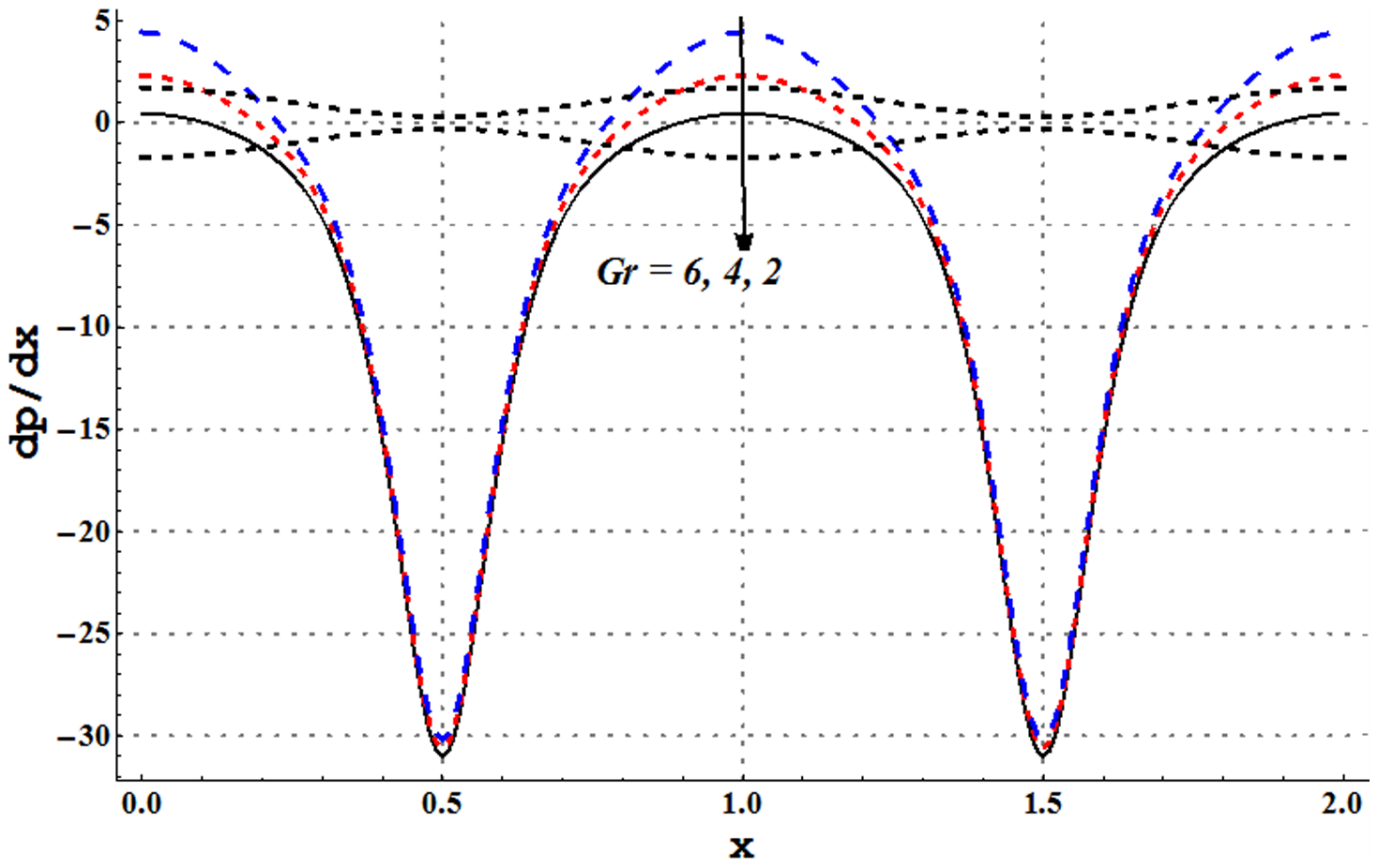
Pressure gradient for variation in Grashoff number when 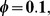 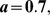 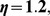 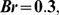 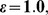 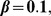 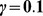 and 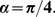

**Figure 5:**
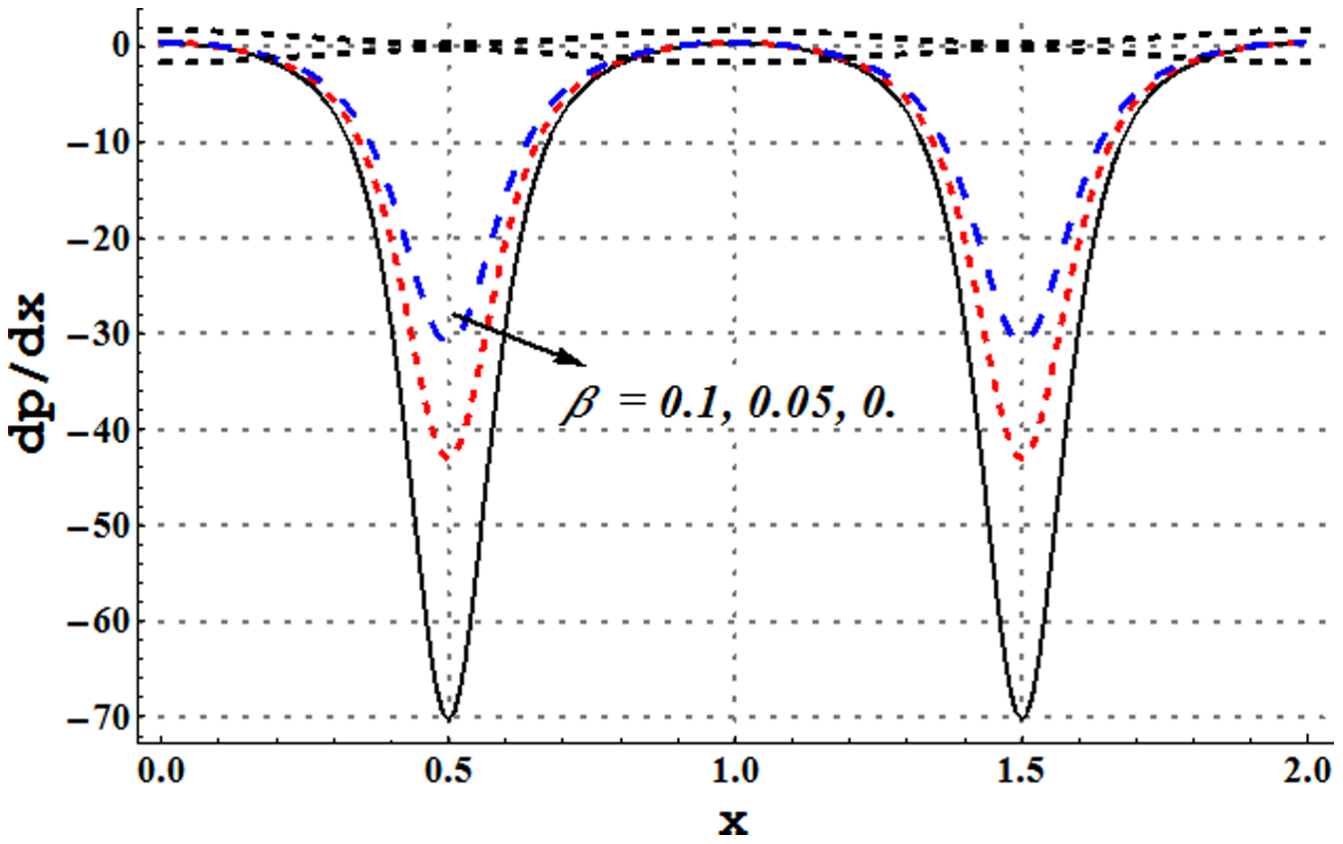
Pressure gradient for variation in velocity slip parameter when 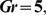 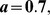 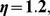 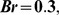 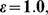 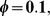 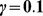 and 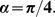

Pressure gradient is studied through Figs. 2–5. These Figs. show that pressure gradient is minimum in the occluded region of channel and attains maximum value in the wider part. Addition of copper nanoparticles reduces the pressure gradient. In addition an increase in the nanoparticle volume fraction decreases the pressure gradient. Such decrease is large in both the wider and occluded regions (see Fig. 2). Fig. 3 depicts that there is an increase in pressure gradient near the wider part of channel when inclinations increases. Effect of Grashoff number on pressure gradient is similar to that of channel inclination angle (see Fig. 4). Pressure gradient enhances when the velocity slip parameter is increased (see Fig. 5). Such increase is large in the occluded portion of the channel.

**Figure 6:**
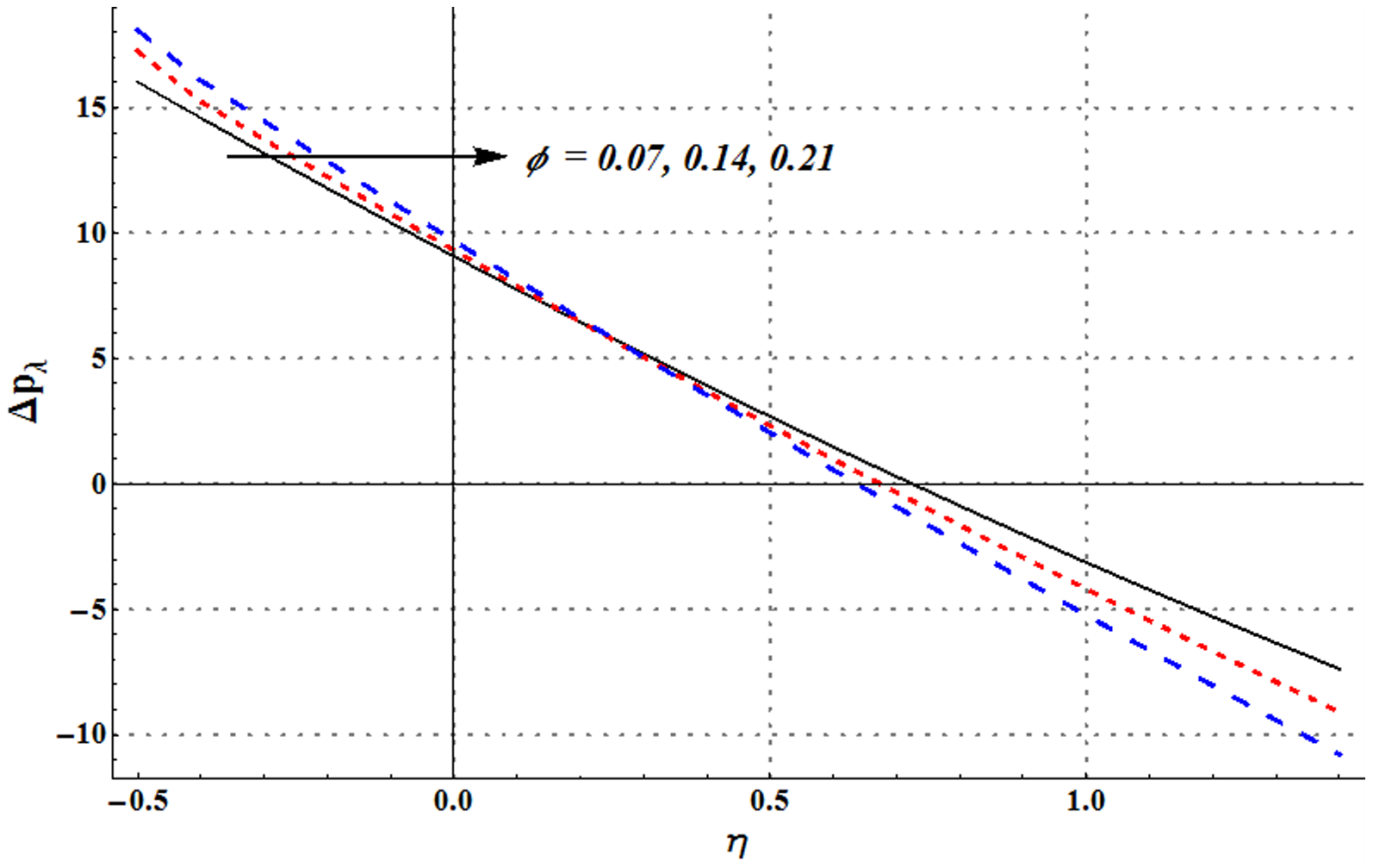
Pressure rise per wavelength for change in nanoparticle volume fraction when 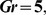 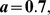 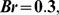 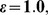 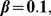 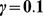 and 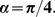

**Figure 7:**
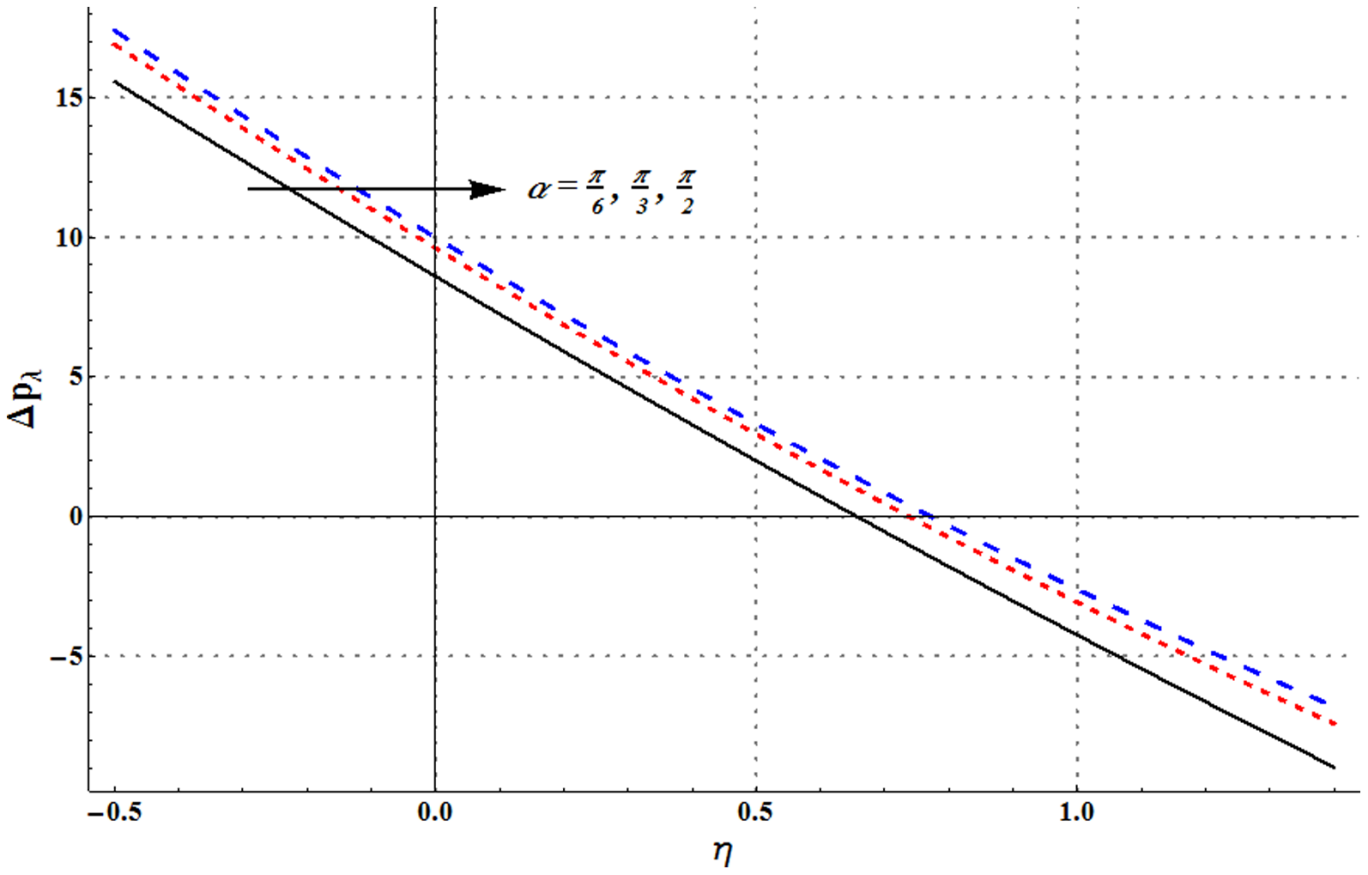
Pressure rise per wavelength for change in channel inclination when 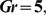 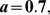 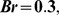 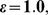 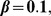 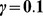 and 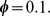

**Figure 8:**
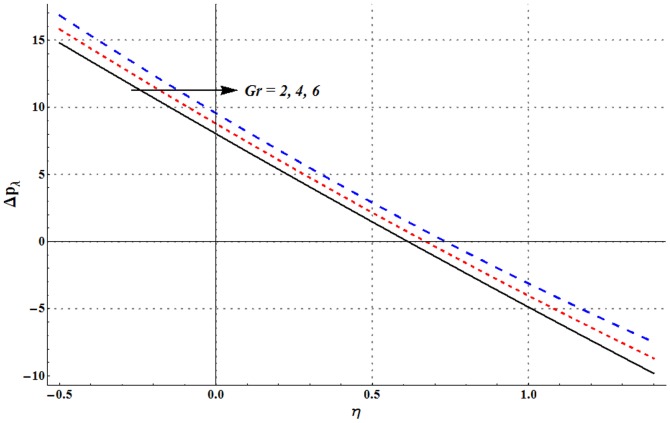
Pressure rise per wavelength for change in Grashoff number when 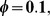 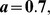 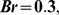 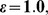 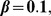 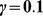 and 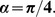

**Figure 9:**
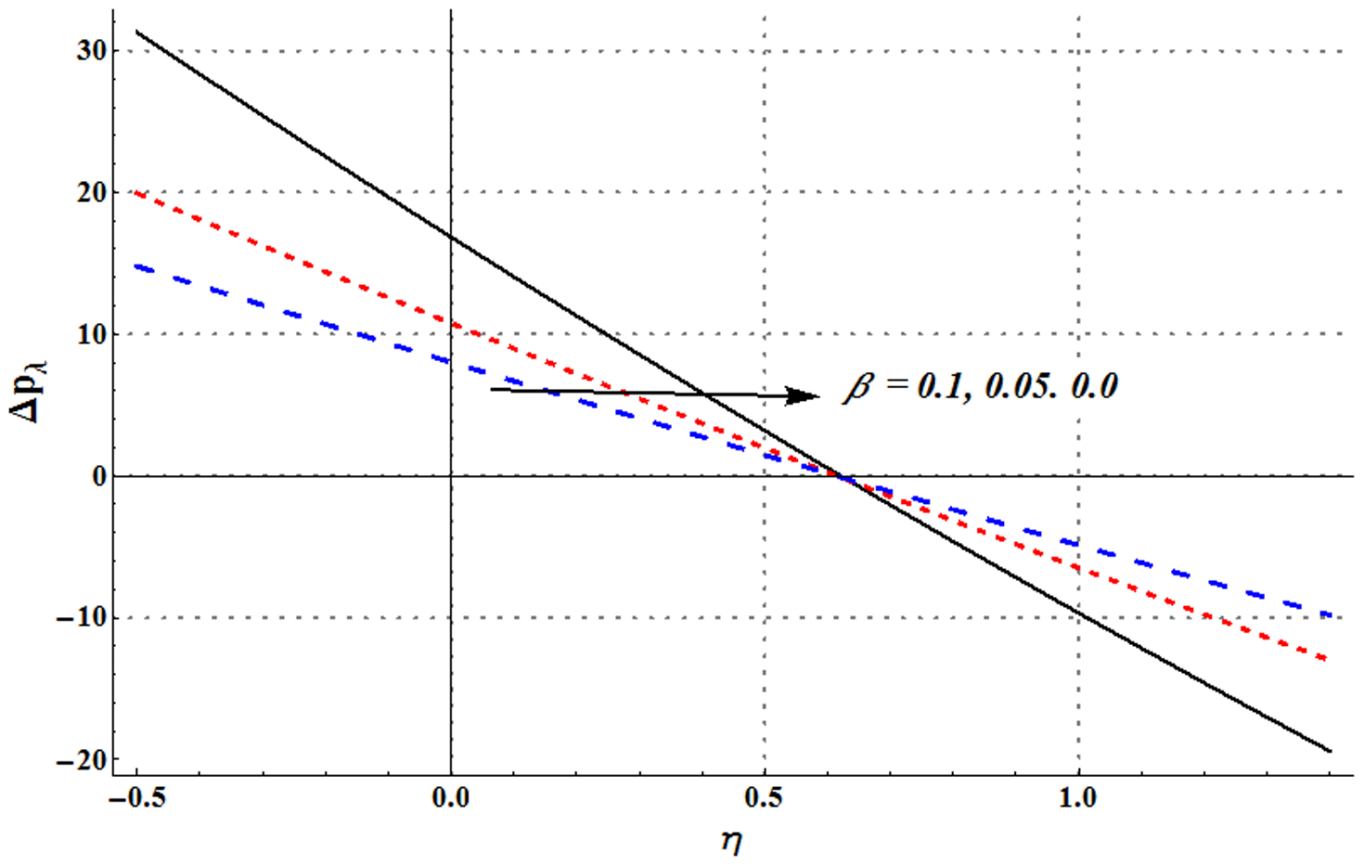
Pressure rise per wavelength for change in velocity slip parameter when 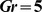, 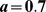, 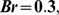 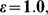 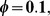 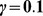 and 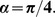

Pressure rise per wavelength versus flow rate is shown in the Figs. 6–9. One common observation from these Figs. is that pressure rise per wavelength decreases with an increase in the flow rate. Fig. 6 shows that addition of nanoparticles decreases the free pumping flux (value of 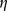 for 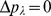). Moreover an increase in the value of nanoparticle volume fraction enhances the pressure rise in retrograde pumping region (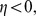 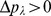). However reverse situation is seen in the augumented pumping region (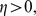 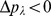). Figs. 7 and 8 show that increase in the values of Grashoff number and channel inclination angle result in the increase of pressure rise per wavelength. Fig. 9 shows that large velocity slip parameter decreases the peristaltic pumping region (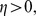 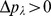). Value of pressure rise per wavelength decreases largely in the retrograde pumping region when 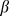 increases.

### Axial velocity and trapping phenomena

**Figure 10:**
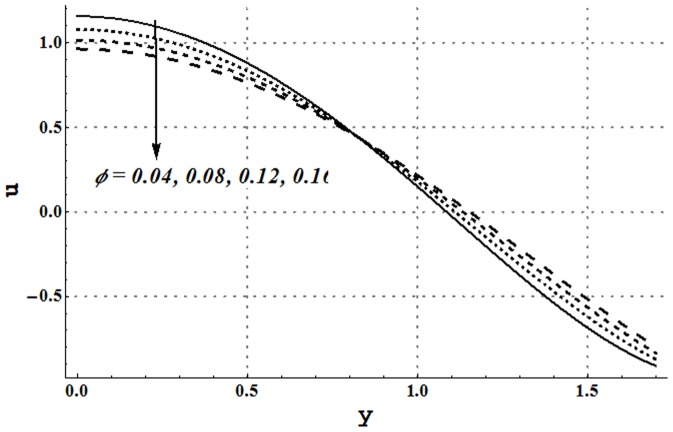
Axial velocity for different nanoparticle volume fraction when 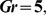 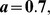 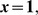 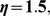 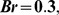 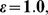 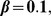 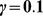 and 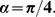

**Figure 11:**
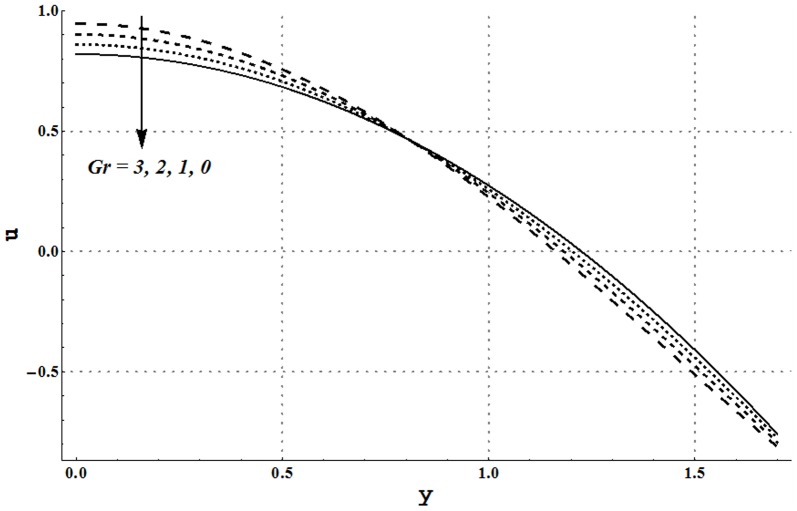
Axial velocity for different Grashoff numbers when 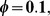 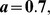 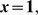 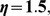 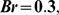 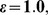 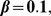 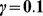 and 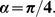

**Figure 12:**
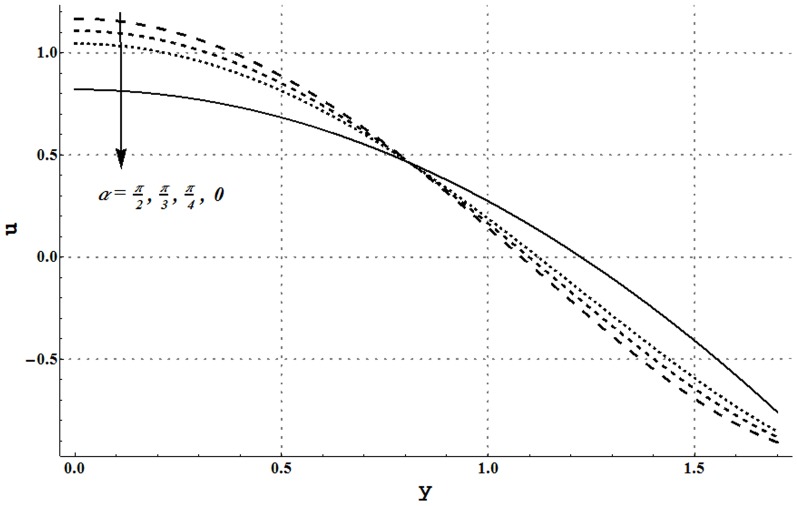
Axial velocity for different inclination angles when 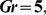 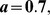 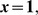 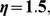 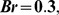 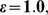 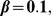 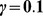 and 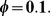

**Figure 13:**
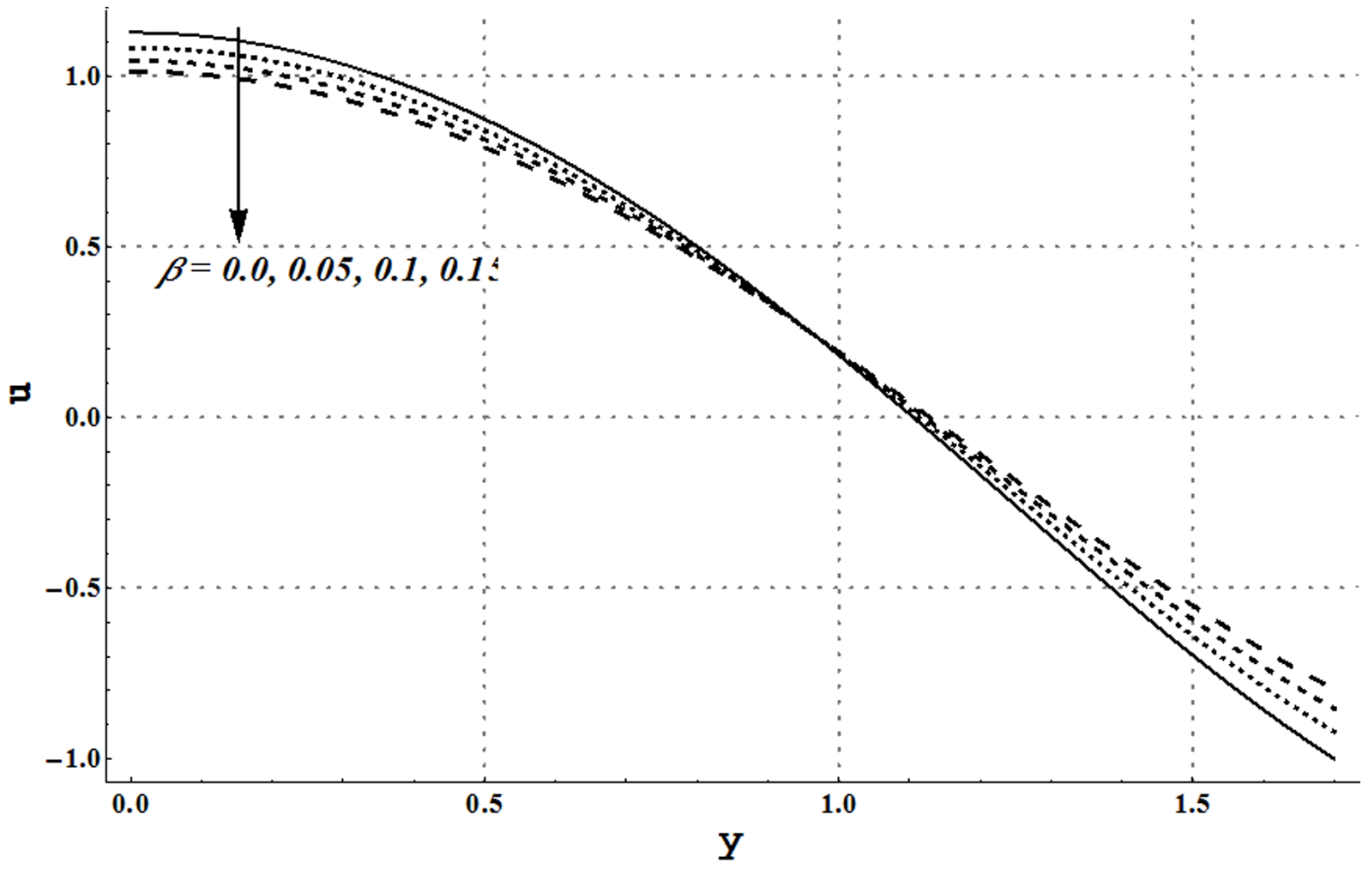
Axial velocity for different values of velocity slip parameter when 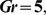 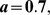 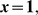 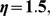 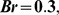 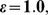 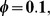 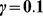 and 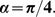

Effects of several flow parameters on axial velocity are shown in Figs. 10–13. These Figs. show that velocity profile traces a parabolic trajectory with maximum value at the center of the channel. Fig. 10 depicts that nanofluid with low concentration of nanoparticles possesses higher value of the velocity at the center. Which concluded that higher nanoparticles volume fraction provides more resistance to the flow. However near the channel walls, this observation is not true which primarily is due to slip effects. Figs. 11 and 12 show that increase in Grashoff number and channel inclination angle result in an increase in velocity at the center. As in previous case this observation is also reversed near the channel walls. Velocity near the channel walls increases with an increase in the velocity slip parameter (see Fig. 13). However maximum value of velocity at the center decreases subject to an increase in 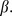


**Figure 14:**
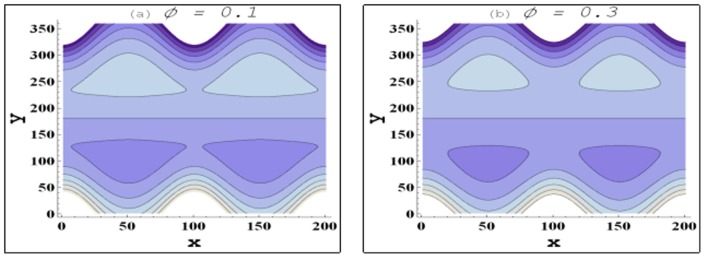
Behavior of streamlines for variation in nanoparticle volume fraction when 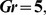 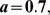 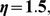 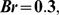 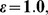 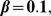 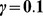 and 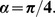

**Figure 15:**
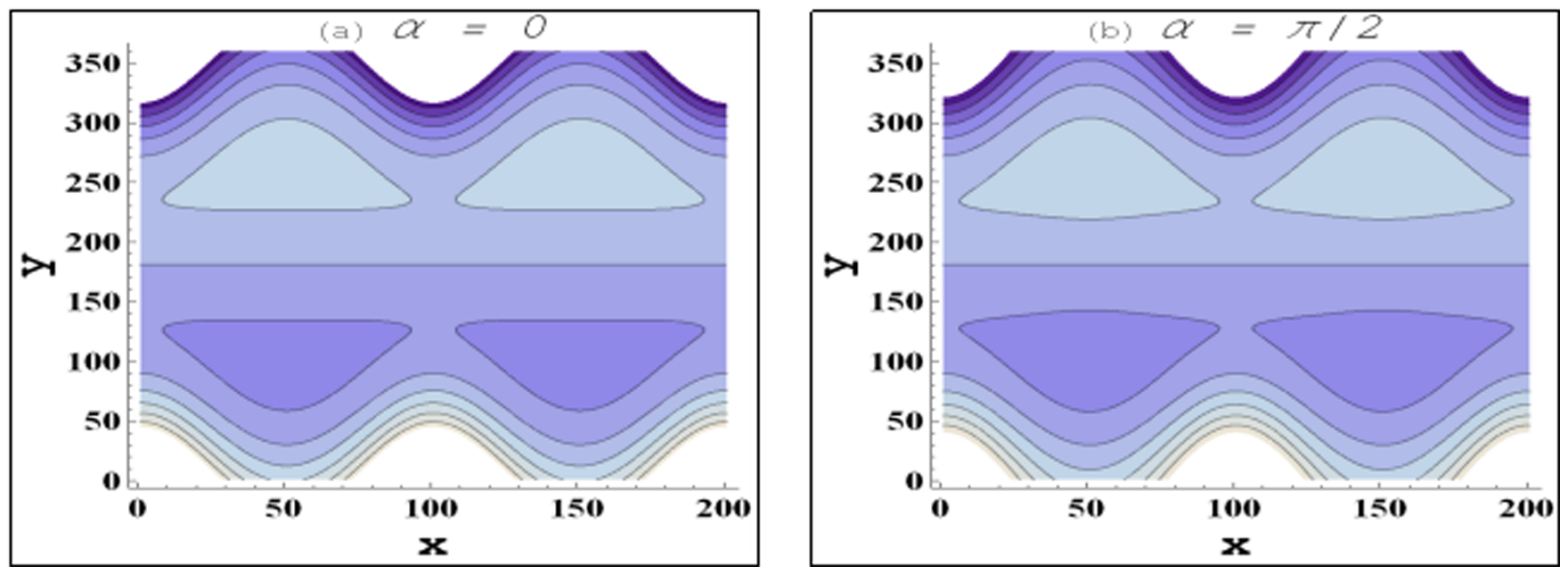
Behavior of streamlines for variation in channel inclination when 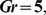 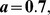 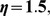 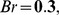 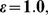 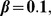 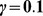 and 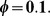

**Figure 16:**
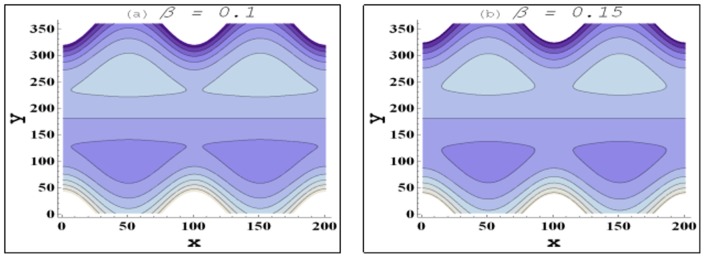
Behavior of streamlines for variation in velocity slip parameter when 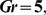 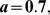 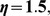 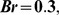 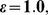 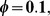 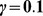 and 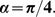

Trapping for different involved parameters is studied through Figs. 14–16. Some volume of fluid during flow tends to get trapped within streamlines. This phenomenon is usually known as trapping and the volume of fluid thus trapped is named as bolus. Size of such bolus reduces with an increase in the value of nanoparticle volume fraction (see Fig. 14). Hence it can be stated that addition of nanoparticles reduces the trapping. Size of the trapped bolus slightly increases with an increase in the channel inclination angle (see Fig. 15). Fig. 16 depicts that size of the trapped bolus reduces with an increase in the velocity slip parameter.

### Heat transfer analysis

**Figure 17:**
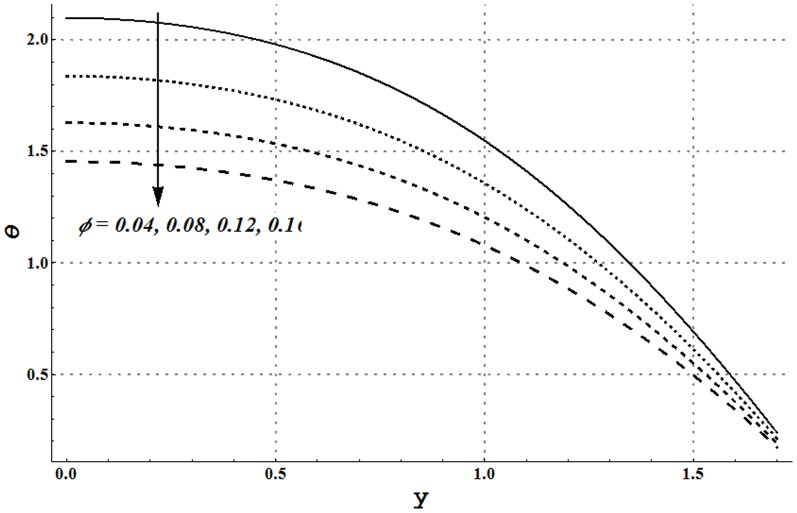
Temperature profile for different nanoparticle volume fractions when 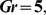 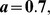 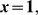 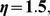 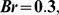 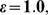 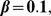 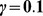 and 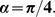

**Figure 18:**
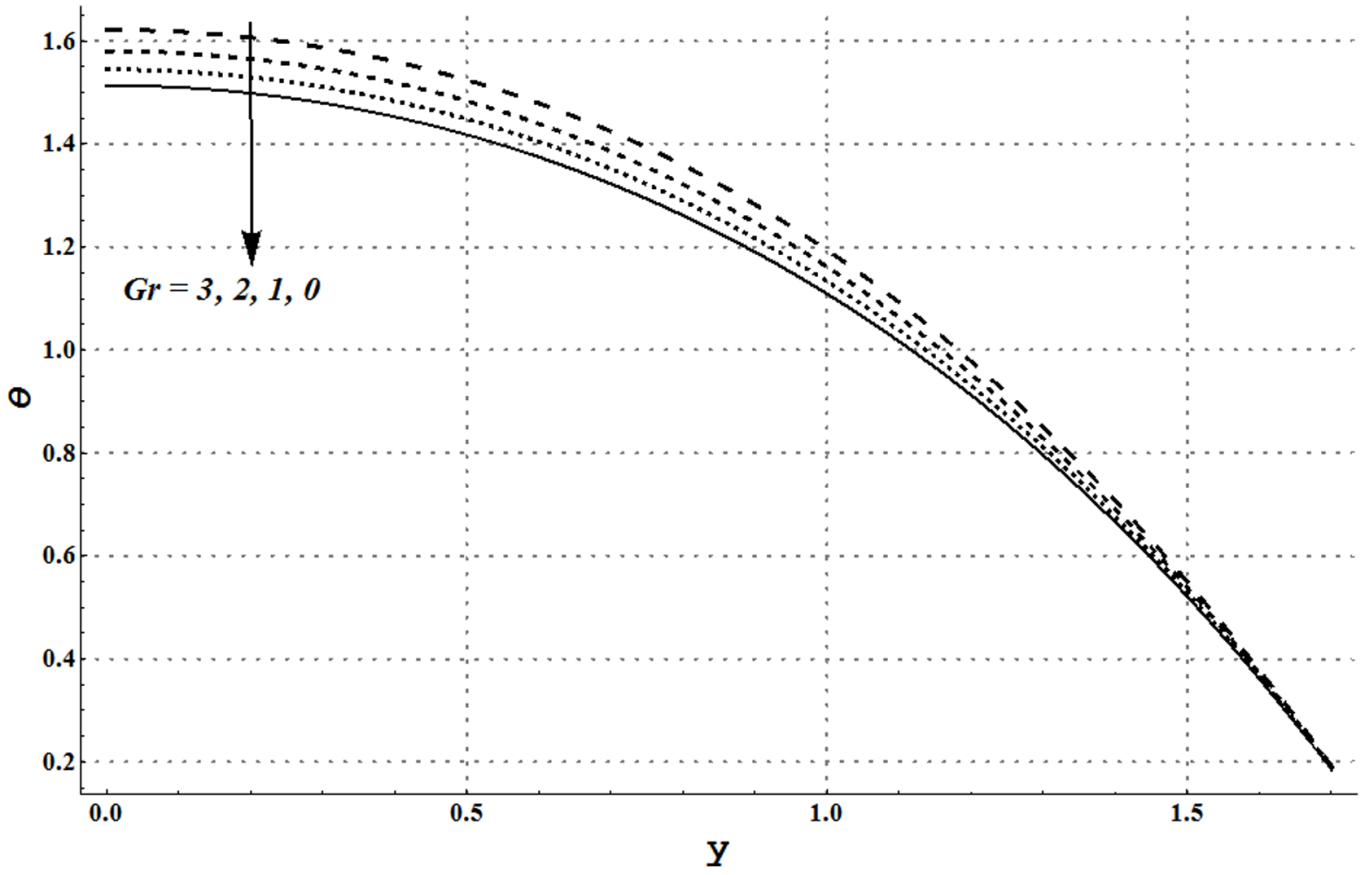
Temperature profile for variation in Grashoff number when 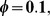 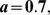 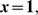 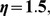 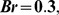 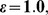 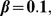 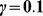 and 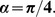

**Figure 19:**
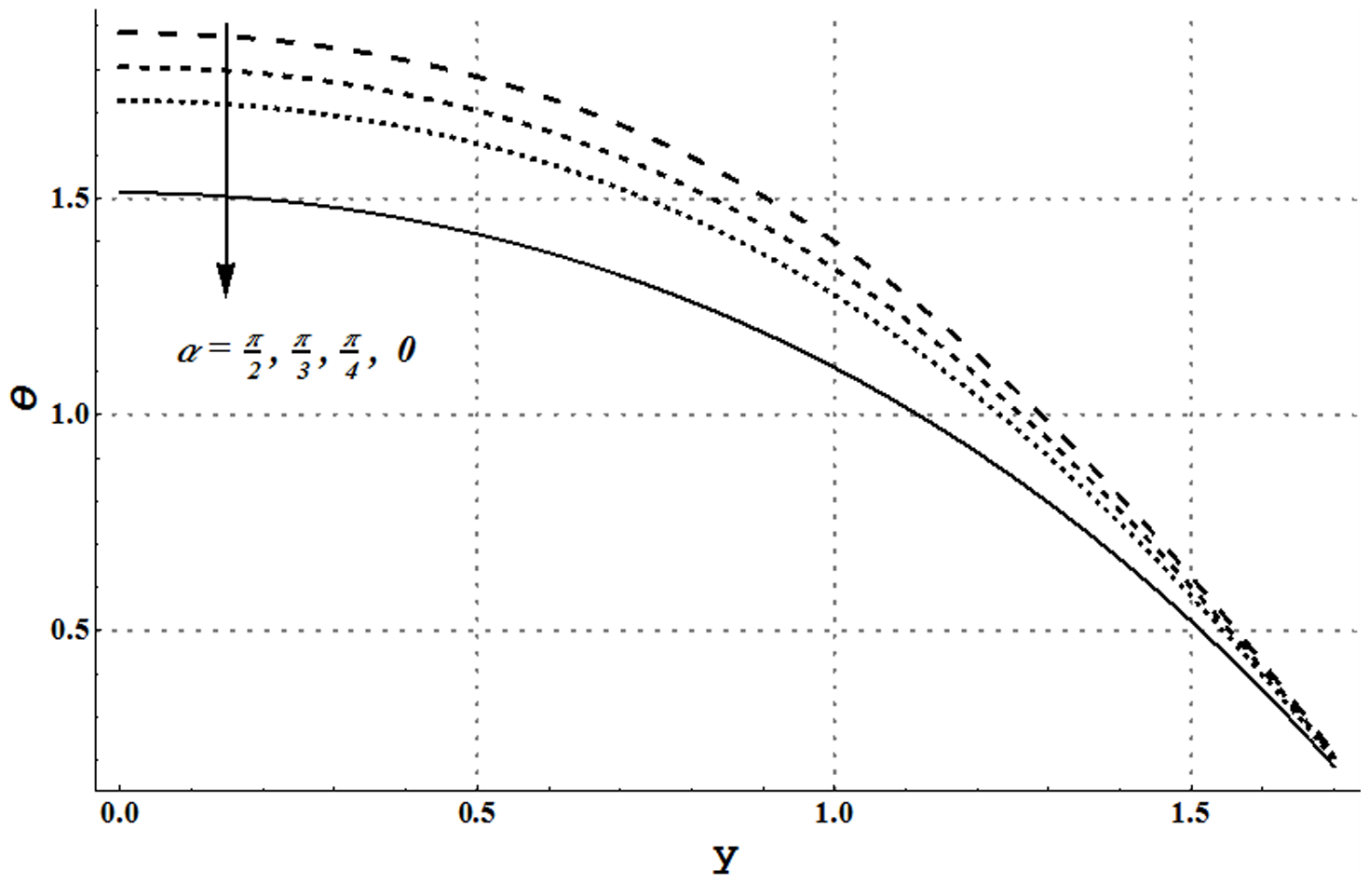
Temperature profile for variation in channel inclination angle when 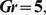 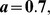 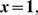 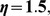 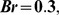 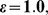 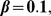 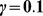 and 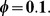

**Figure 20:**
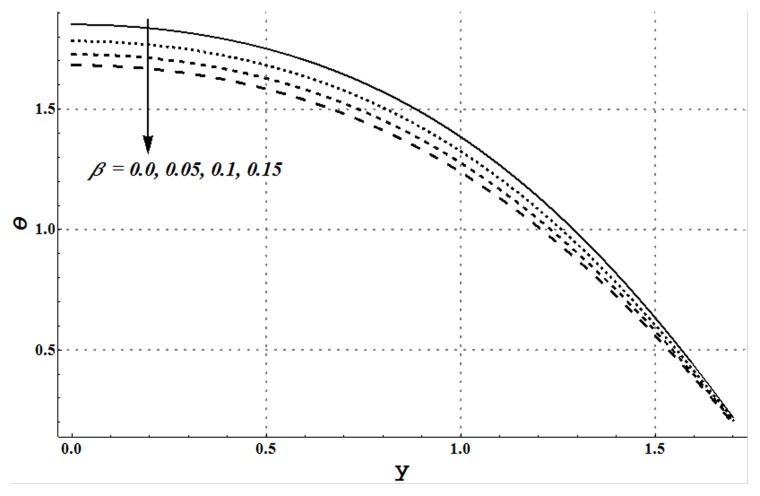
Temperature profile for variation in velocity slip parameter when 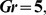 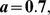 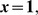 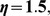 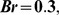 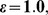 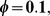 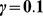 and 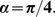

**Figure 21:**
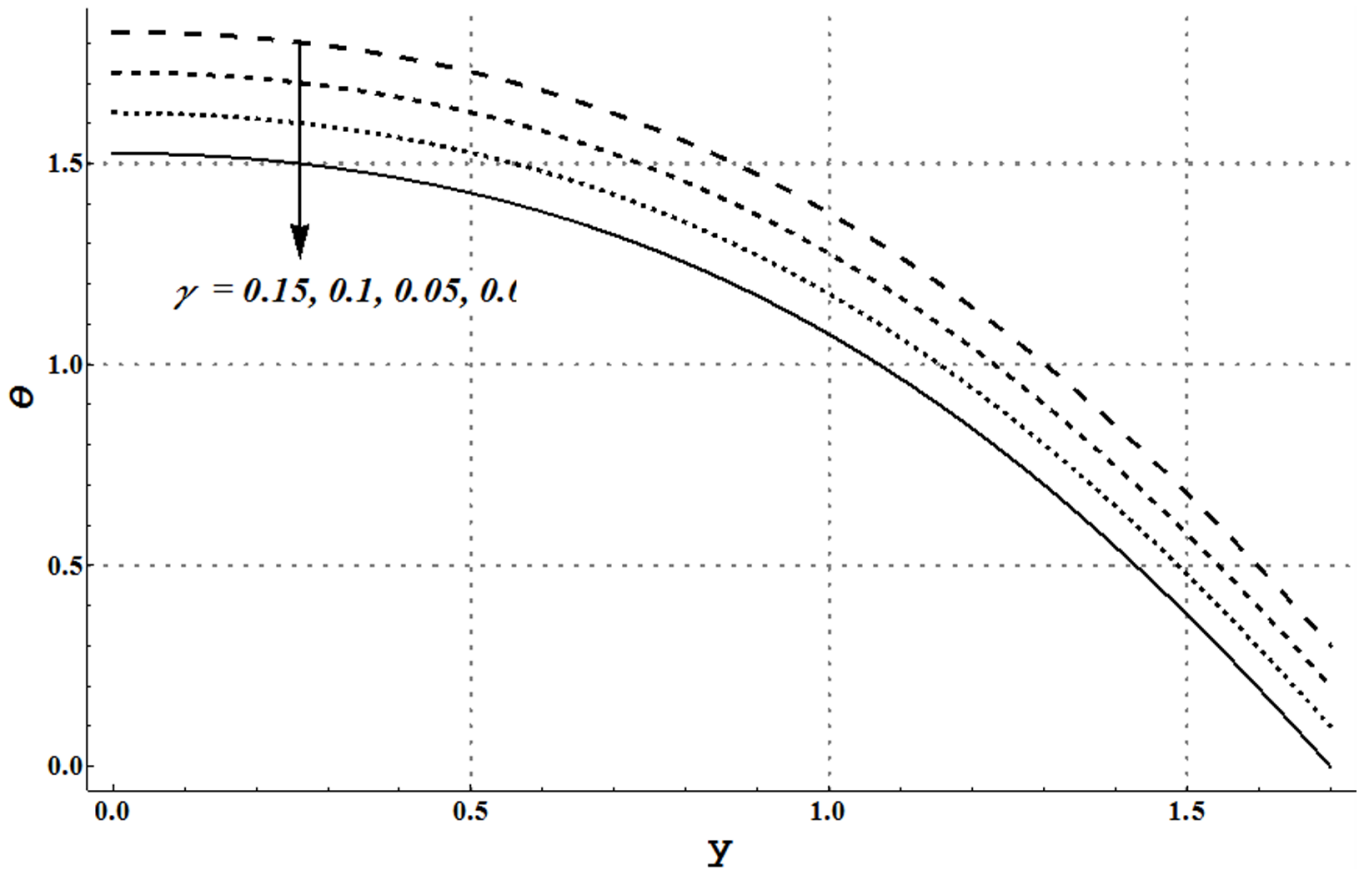
Temperature profile for variation in thermal slip parameter when 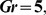 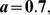 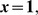 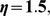 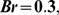 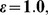 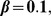 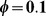 and 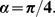

**Figure 22:**
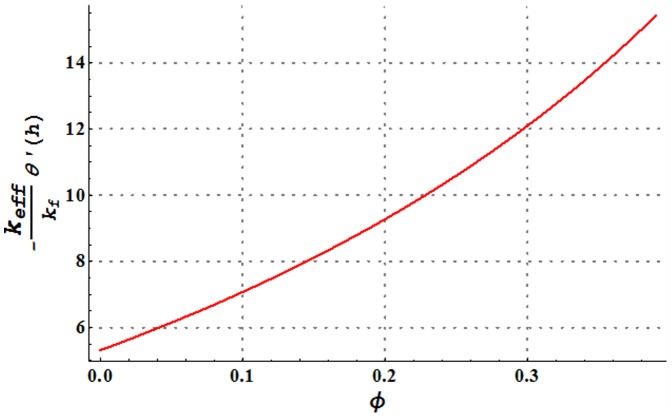
Change in heat transfer rate at the wall for variation in nanoparticle volume fraction 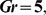 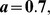 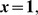 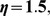 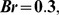 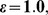 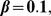 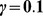 and 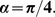

**Figure 23:**
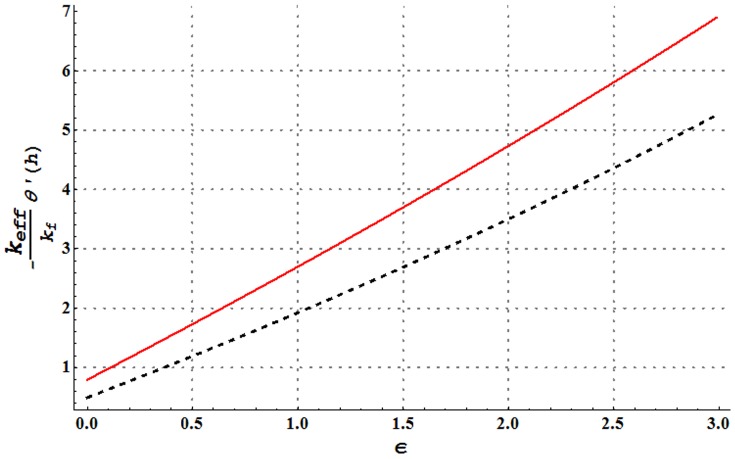
Comparison of heat transfer rate at the wall of water and Cu-water nanofluid for change in heat source/sink parameter when 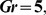 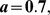 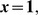 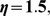 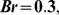 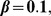 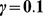 and 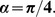

**Figure 24:**
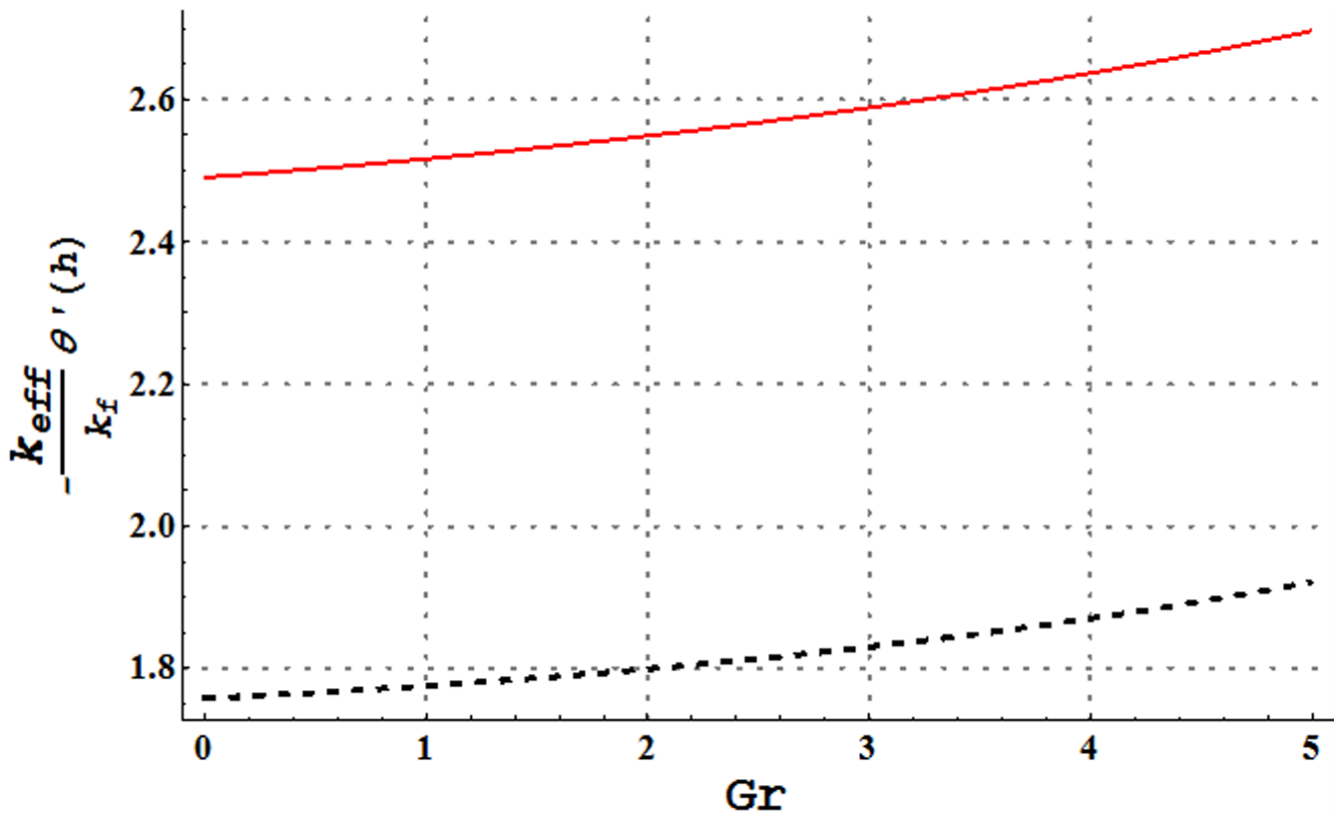
Comparison of heat transfer rate at the wall of water and Cu-water nanofluid for change in Grashoff number when 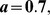 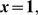 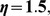 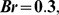 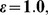 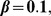 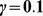 and 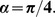

**Figure 25:**
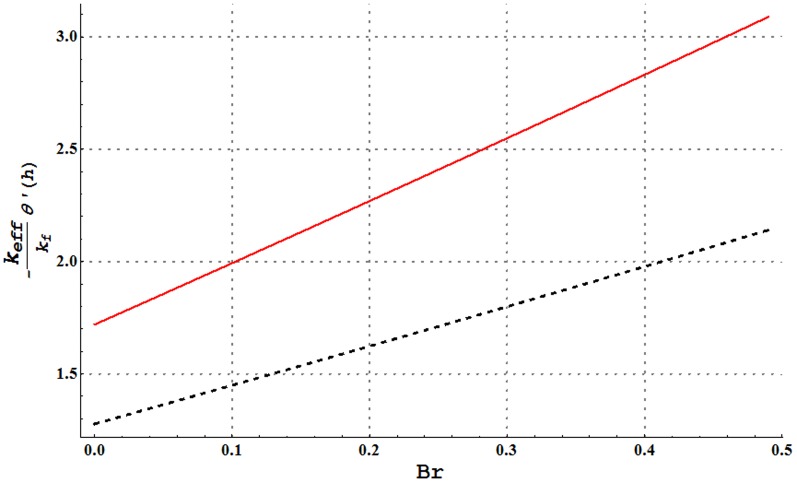
Comparison of heat transfer rate at the wall of water and Cu-water nanofluid for change in Brinkman number when 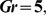 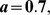 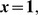 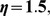 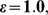 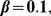 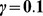 and 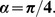

Impact of several embedded parameters on temperature and heat transfer rate at the wall are examined through Figs. 17–25. It is clear from Fig. 17 that an increase in the nanoparticle volume fraction shows a decrease in fluid temperature. This clearly witnesses that the heat being generated by the heat source is quickly transferred to the walls (due to addition of more nanoparticles) and consequently the fluid temperature decreases. High thermal conductivity of nanoparticles plays a key role in quick dissipation of the fluid temperature. This justifies the use of copper nanoparticles in different types as the coolants. Figs. 18 and 19 show that increase in the values of Grashoff number and channel inclination enhance the fluid temperature. This is due to the fact that increase in *Gr* and 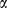 facilitates the convective heat transfer within the fluid. Figs. 20 and 21 show that velocity and thermal slip parameters have opposite effects on temperature of cu-water nanofluid. Temperature of nanofluid decreases with an increase in the velocity slip parameter.

Heat transfer rate at the wall for variation in nanoparticle volume fraction is analyzed in Fig. 22. It is observed that larger nanoparticle volume fraction increases the heat transfer rate at the wall. Hence addition of copper nanoparticles facilitates the heat transfer between the fluid and solid boundary and ultimately preventing the fluid from heating. Figs. 23–25 present a comparison of the heat transfer rate at the wall of water in the absence and presence of copper nanoparticles. All these Figs. show that copper-water nanofluid possesses higher heat transfer rate at the wall when compared with ordinary water. Also heat transfer rate is seen to increase by increasing 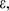 *Gr* and *Br*. Numerical values of heat transfer rate at the wall for variations in 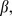 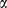 and 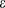 are given in Table 3. It is observed that heat transfer rate at the wall increases when 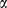 and 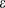 are increased. However it decreases through larger 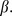 Comparison between the limiting case of present study and the analysis performed by Ali et al. [28] is provided through Table 4.Critical values of flow rate are provided and compared. Good comparison is found between the two.

**Table 3:** Numerical values of heat transfer rate at the wall for change in the values of different parameters when 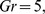 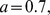 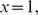 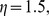 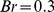 and 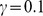.

*β*	*α*	*ε*	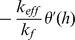
0.0	*π*/4	1.0	2.93204
0.05			2.79028
0.10			2.67728
0.15			2.58574
0.10	*π*/6		2.46308
	*π*/4		2.67727
	*π*/3		2.76587
	*π*/2		2.86097
	*π*/4	0.0	0.78554
		1.0	2.67227
		2.0	4.70860
		3.0	4.90948

**Table 4:** Comparison of present study with the results obtained by obtained by Ali et al [28] when 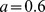 and 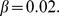

	Alit et al. [28]	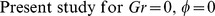
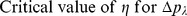	0.45022	0.45233405
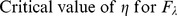	0.3501	0.3505850

## Conclusions

Velocity and thermal slip effects on the peristaltic transport of copper-water nanofluid in an inclined channel are studied. Findings of present analysis indicate that the addition of copper nanoparticles reduces the pressure gradient. Increase in the value of nanoparticle volume fraction enhances the pressure rise in retrograde pumping region. Nanofluid with low concentration of copper nanoparticles possesses higher value of velocity at the center of channel. Velocity slip parameter has a decreasing effect on the maximum value of axial velocity. Trapping is reduced in presence of nanoparticles. Grashoff number and channel inclination angle have increasing effect on the temperature of nanofluid. Increase in nanoparticle volume fraction largely enhances the heat transfer rate at the wall.
